# Ewing sarcoma of the uterus: A case report

**DOI:** 10.1016/j.crwh.2024.e00640

**Published:** 2024-07-23

**Authors:** Zeynep Tek, Anya Laibangyang, Oluwole Odujoko, Bhavna Khandpur, David Doo

**Affiliations:** a Department of Obstetrics and Gynecology, Danbury and Norwalk Hospital, Nuvance Health, United States; b Department of Pathology, Danbury and Norwalk Hospital, Nuvance Health, United States

**Keywords:** Ewing sarcoma of the uterus, Primary neuroendocrine tumors, Rare tumors of the female urogenital tract

## Abstract

A case is described of Ewing sarcoma of the uterus, an atypical presentation of an already rare cancer.

A 55-year-old woman presented with abdominal pain, abnormal uterine bleeding and a uterine mass that measured 11 × 10 × 14.5 cm and demonstrated heterogeneous enhancement with possible areas of central necrosis, concerning for sarcoma. She had a complete surgical resection with total abdominal hysterectomy, bilateral salpingo-oophorectomy, omentectomy, bilateral pelvic lymph node dissection, and excision of mesenteric tumor implants. Her final pathology showed primary Ewing sarcoma-primitive neuroectodermal tumor of the uterus with metastatic spread to the peritoneal cavity. She finished 14 cycles of vincristine-doxyrubicin-cyclophosphamide–ifosfamide, etoposide chemotherapy with no evidence of recurrent metastatic disease at 6-month follow-up.

Ewing sarcoma is a rare cancer, predominantly seen in adolescents, that typically are of the bone, although in rare instances it can arise from soft tissue; even rarer are presentations in the female genital tract. Even with typical presentations of Ewing sarcoma of the bone, metastatic disease has an overall poor prognosis. The scarcity of cases of metastatic Ewing sarcoma–peripheral neuroendocrine tumors of the uterus makes the condition especially difficult to study. This report describes a case of Ewing sarcoma of the uterus treated by complete surgical resection and aggressive multimodal chemotherapy.

## Highlights

• Ewing sarcoma of the uterus is an atypical presentation of an already rare cancer.
• The incidence of extraosseous sites of Ewing sarcoma is 0.4/1,000,000.
• Due to the rarity of neuroendocrine tumors of the gynecologic tract, it is difficult to histologically differentiate between central and peripheral primary neuroendocrine tumors.
• There is no set standard of care for primary neuroendocrine tumors in the uterus, but treatment protocols might differ between central- and peripheral-type tumors.
• In the case reported, patient was treated with full surgical excision and vincristine/doxorubicin/cyclophosphamide–ifosfamide/etoposide (VDC-IE) every 2 weeks for 14–17 cycles.

## 1 Introduction

Ewing sarcoma is a type of primitive neuroectodermal tumor and is the second most common malignancy of the bone. It most commonly presents in the second decade of life, with an incidence of 9–10 per million worldwide [1]. It is classified as a peripheral primary neuroendocrine tumor (p-PNET). It is similar in histology to central primary neuroendocrine tumors; however, the changes in morphology can have a significant effect on long-term survival. The use of chemotherapy has overall improved the average 5-year survival rate of patients with p-PNET from 10% to 70–80%, with the current standard of care being vincristine, doxorubicin, cyclophosphamide–ifosfamide, etoposide (VDC–IE) every 2 weeks for 14 cycles [2]. Unfortunately, prognosis is poor for patients with metastatic disease, with a 5-year survival as low as 15–30%. Non-metastatic primary soft-tissue sites of the tumor account for 20–30% of newly diagnosed cases, with an incidence of 0.4/1,000,000 in the United States. Most commonly, extraosseous sites include the upper thigh, buttocks and arms. It is extremely rare in the female genital tract, with only 40 cases reported in the literature between 1970 and 2023. Due to the rarity of the disease in the female genital tract, the diagnosis is difficult to make on both preoperative imaging as well as final pathology and best practices for treatment are still unknown. The case reported here is an example of a rare presentation of metastatic Ewing sarcoma of the uterus in a 55-year-old woman.

## 2 Case Presentation

**Fig. 1:**
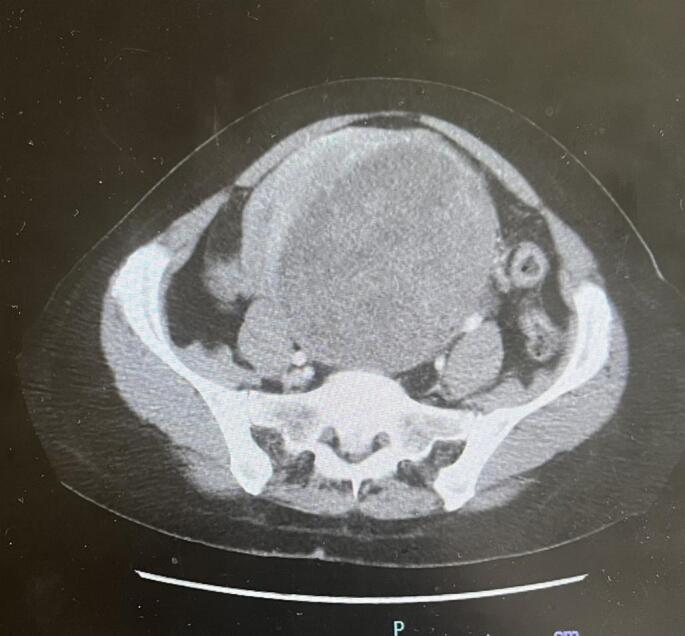
A mass from the left uterus measuring 11 × 10 × 14.5 cm, which demonstrates heterogeneous enhancement with possible central necrosis areas.

A 55-year-old woman, G3P3003, with a past medical history of psoriasis and a past surgical history of two prior cesarean deliveries, originally presented to the emergency department with newfound abdominal pain for 3 days, and 6 months of worsening menorrhagia and constipation. CT of the abdomen/pelvis showed a 11x10x14.5 cm mass demonstrating heterogenous enhancement with possible areas of central necrosis, with concern for uterine leiomyosarcoma (Fig. 1). Gynecology oncology was consulted at the time with plans for immediate surgical management. The patient underwent a total abdominal hysterectomy, bilateral salpingo-oophorectomy, omentectomy, bilateral pelvic lymph node dissection, and excision of peritoneal mesenteric tumor implants. She was noted to have extensive adhesive disease secondary to her previous cesarian deliveries and from her tumor, as well as anatomical distortion due to the presence of the large tumor.

**Fig. 2:**
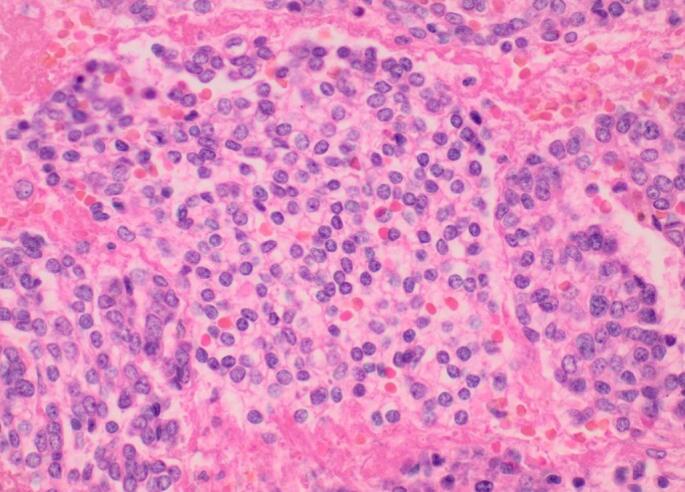
Sections show the tumor cells at high magnification with characteristic clearing of the cytoplasm. (H&E X40).

**Fig. 3:**
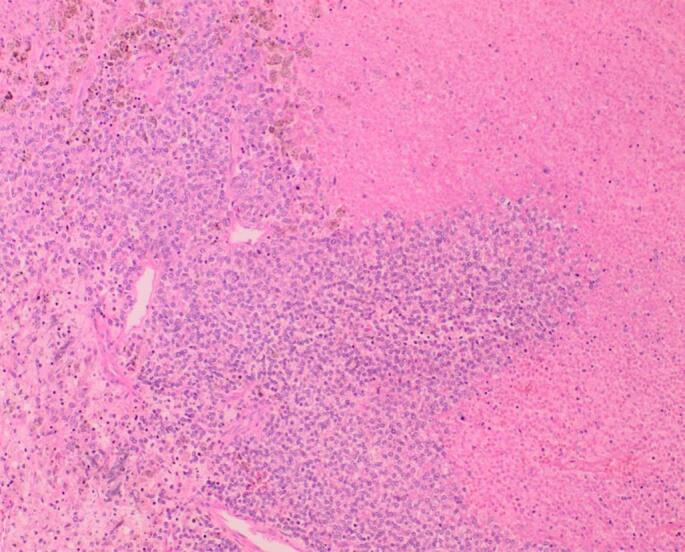
Sections show tumor with areas of extensive necrosis at the right corner (blue arrow).(H&E X10). (For interpretation of the references to colour in this figure legend, the reader is referred to the web version of this article.)

The pathology report described extensive necrosis and focal hemorrhage, with hemosiderin pigment present. Immunostains were positive for vimentin and cyclin D1, and negative for CD 10, ER, PR CK1/3, CK7, CK20, PAX8, LCA, Actin, SMMHC, HMB45, CD117, Desmin and Caldesmon. A histologically similar neoplasm was found in the mesenteric implants. Her cervix, fallopian tubes, ovaries, and bilateral lymph nodes were normal (Fig. 2, Fig. 3). Due to the nonspecific immunostaining with the unclear diagnostic picture, the case was sent for outside review for consultation. Pathology review found primitive neuroectodermal tumor peripheral type, with uterine tumor burden extending to the posterior uterine serosal defect. Archer Fusion Plex testing showed the presence of an EWSR1-FLI1 rearrangement confirming the diagnosis of p-PNET.

Following surgery, she began adjuvant VDC–IE chemotherapy. A PET scan showed slightly increased portal hepatic FDG uptake and new moderate FDG uptake in the portocaval lymph node. She was offered extended beam pelvic radiation but declined due to the side-effects and finished 14 cycles of VDC–IE chemotherapy. A repeat CT scan of the chest and MRI of the abdomen and pelvis found no evidence of metastatic disease and no new suspicious findings. Plans were made to continue routine surveillance every 3 months.

## 3 Discussion

Primary neuroectodermal tumors are thought to be derived from fetal neuroectodermal cells, with morphologic features of small round cells of the central nervous system [3]. EWSR1 rearrangement mutations are indicative of Ewing sarcoma, or peripheral PNET. Due to the rarity of PNET tumors in the gynecologic tract, and the similarities in histology between peripheral and central PNET tumors (c-PNET), a distinction is often not made [3]. Many of the cases of uterine PNET tumors reported in the literature lack the EWSR1 gene translocation, and therefore resemble c-PNET [4]. In a literature review looking at 19 different case reports of PNET in the female genital tract, only 4 were p-PNETs with the EWSR1 rearrangement (3).

Understanding the distinction between central and peripheral PNETs is important for clinical treatment due to the known chemotherapy regimens for Ewing sarcoma. The Children's Cancer Group Pediatric oncology group showed that ifosfamide and etoposide, alternating with vincristine, doxorubicin, and cyclophosphamide for 17 cycles improved overall and event-free survival in patients with localized tumors [5]. Although this regimen has proven to be less effective with metastatic Ewing sarcoma of the bone, it is part of the standard of care. C-PNET tumors utilize a separate chemotherapy regimen, traditionally a combination involving vincristine, cisplatinum, cyclophosphamide, and etoposide [6].

There is no standard of care for management of Ewing sarcoma of the uterus. Surgery with complete resection is the most well documented therapy [[7], [8], [9], [10]]. After surgical resection, post-operative chemoradiation is considered; however, there is no current agreed best practice. VDC-IE chemotherapy is the chemotherapy protocol approved for bone origin of Ewing sarcoma and was the protocol in our case. The adjuvant VDC-IE regimen was used consistently across case reports; however, the length of the cycles varied, most commonly between 6 and 9 cycles. Prognosis for stage 1 A of the disease is excellent, but with metastatic disease the average disease-free interval is 10–18 months [7,11].

In the present case, the patient had complete surgical resection and her chemotherapy regimen was identical to that of Ewing sarcoma of the bone: 14 cycles of VDC-IE. The patient was offered full pelvic radiation but declined due to the side-effect profile. On her CT scan of the chest and MRI of the abdomen/pelvis over 12 months later she continued to remain without any new interval growth. Due to the rarity of the tumor, the optimal treatment regimen is unknown, but complete surgical resection is recommended if possible, due to the known resistance to chemotherapy of this tumor type.
